# Extant Genus in the Mesozoic: *Paleoplatyura* Meunier (Diptera: Keroplatidae) Found in the Cretaceous Amber of Myanmar †

**DOI:** 10.3390/insects13010024

**Published:** 2021-12-24

**Authors:** Jan Ševčík, Wiesław Krzemiński, Kornelia Skibińska

**Affiliations:** 1 Department of Biology and Ecology, Faculty of Science, University of Ostrava, Chittussiho 10, 710 00 Ostrava, Czech Republic; sevcikjan@hotmail.com; 2 Institute of Systematics and Evolution of Animals Polish Academy of Sciences, 31-016 Kraków, Poland; wieslawk4@gmail.com

**Keywords:** fossil insects, Sciaroidea, Bibionomorpha, inclusions, Mesozoic, taxonomy

## Abstract

**Simple Summary:**

Burmese amber is very rich in perfectly preserved insects. Consequently, it is an invaluable source of information for taxonomic and evolutionary studies. Moreover, it forms a unique connection between the Jurassic and Cretaceousfaunas and documents the first representatives of modern genera. In this paper, a primitive genus of Keroplatidae, *Paleoplatyura* Meunier, 1899, is recorded from Burmese amber for the first time. This represents a rather rare case of the presence of an extant insect genus in the Mesozoic. Three new species of *Paleoplatyura* are described, indicating that this genus was relatively diverse already in the Cretaceous.

**Abstract:**

Three new species of *Paleoplatyura* Meunier, 1899, i.e., *Paleoplatyura agnieszkae* sp. nov., *P. miae* sp. nov., and *P. magnifica* sp. nov., are described and figured. The concept of the genus is briefly discussed, and its systematic position is clarified. A key to fossil species is provided. The genus *Paleoplatyura* is described from the Eocene Baltic amber. It is concluded that, in Baltic amber, this group is represented only by the type species, and the identity of the other two species is problematic. No additional specimens have been found so far in this amber. Therefore, the presence of as many as three new species in Burmese amber, certainly belonging to *Paleoplatyura*, is a confirmation of its occurrence already in the Mesozoic.

## 1. Introduction

The fossil record of the family Keroplatidae (Diptera: Bibionomorpha) is still relatively scarce, especially from the Mesozoic, with only several taxa formally described [1,2]. This family currently comprises six extant subfamilies, Arachnocampinae Matile, 1981 [3], Keroplatinae Rondani, 1856 [4], Lygistorrhininae Edwards, 1925 [5], Macrocerinae Rondani, 1856 [4], Platyurinae Loew, 1850 [6], Sciarokeroplatinae Papp and Ševčík, 2005 [7], and one fossil subfamily, Adamacrocerinae Ševčík, Krzemiński and Skibińska, 2020 [2], from the mid-Cretaceous Burmese amber. Unfortunately, the limited number of clear morphological criteria defining some of the subfamilies of Keroplatidae, as well as the absence of a unique synapomorphy of the family [1,2] and the still widely discussed phylogenetic relationships within Sciaroidea [8], make it difficult to classify new fossil taxa of Keroplatidae to particular subfamilies. A typical example represents the genus *Paleoplatyura* Meunier, 1899 [9]. This genus has traditionally been considered to belong to the subfamily Keroplatinae, in the tribe Orfeliini Matile, 1990 [10] (e.g., Matile [10]); although, some authors (e.g., Shaw [11]) pointed out that *Paleoplatyura* shows the most plesiomorphic wing venation among fungus gnats (Sciaroidea) and can be considered as a “living fossil” [12]. Mantič et al. [1] reinstated and redefined a separate subfamily Platyurinae Loew, 1850 [6], for the Palearctic species *Platyura marginata* Meigen, 1803 [13], and the Nearctic *Platyura pectoralis* Coquillett, 1895 [14], and *Paleoplatyura melanderi* Fisher, 1941 [15] (the latter species not considered by them as true *Paleoplatyura*), while *Paleoplatyura johnsoni* Johannsen, 1910 [16], was classified as Keroplatidae incertae sedis, in a well-supported clade of various genera more or less related to Macrocerinae. 

The genus *Paleoplatyura* currently formally includes three extant species, i.e., *P. aldrichii* Johannsen, 1909 [17]; *P. johnsoni* Johannsen, 1910 [16], and *P. melanderi* Fisher, 1941 [15], and three described fossil species, i.e., *P. macrocera* (Loew, 1850 [6]), *P. loewi* Meunier, 1922 [18], and *P. (?) eocenica* Cockerell, 1921 [19]. However, the placement of *P. aldrichii*, *P. melanderi*, *P. loewi* and *P. eocenica* in the genus *Paleoplatyura* is questionable and most probably wrong. The holotype of *P. aldrichii* is probably lost [20], and the original description by Johannsen [17] is very short, without any figure provided, only Johannsen [16] mentioned that “this species differs in several important structural characters from *P. johnsoni*” (he explicitly specifies only the subcostal cross-vein absent and cubital vein not reaching wing margin), indicating that *P. aldrichii* probably does not belong to true *Paleoplatyura*. Concerning *P. melanderi*, Mantič et al. [1] did not consider this species as true *Paleoplatyura*, because of R_2+3_ ending in R_1_ and its considerable molecular distance from *P. johnsoni*. Additionally, *P. loewi* does not belong to *Paleoplatyura*, because this species lacks the transverse r-m vein characteristic for this genus and possesses the more apomorphic r-m fusion, typical of most keroplatids. This can be seen exactly in the drawing of the wing given in the work of Meunier [18], p. 3, Figure 1., where the vein M_1+2_ merges at some distance with the vein Rs. The identity of *P. eocenica* is most obscure, because nothing important can be inferred from the original description and it is even unclear if the species belongs to Keroplatidae. Cockerell [19] himself was not sure whether this species belongs to the genus *Paleoplatyura*, providing a question mark after the genus name in the original description. 

In this paper, we aim to clarify the taxonomic concept of the genus *Paleoplatyura* and describe three new Cretaceous species of this remarkable keroplatid genus. 

## 2. Materials and Methods

Specimens were examined using a Nikon (Minato, Japan) SMZ25 stereomicroscope, equipped with a Nikon DS-Ri2 digital camera. Photomicrographs are focus stacks captured using this system and processed using NIS-Elements Imaging Software (Minato, Japan). Line drawings were produced by tracing photographs. The terminology follows Ševčík et al. [21], where the homology and wing vein nomenclature in Bibionomorpha are briefly explained. The holotypes are deposited in the collection of the Institute of Systematic and Evolution of Animals Polish Academy of Sciences (ISEA PAS) and paratypes in the National Museum, Prague, Czech Republic (NMPC). The specimens described here come from the Hukawng Valley in Kachin State, northern Myanmar. Burmese amber was dated by Cruickshank and Ko [22] to the middle–late Albian, based on insect inclusions and a specimen of the ammonite, but Grimaldi et al. [23] estimated the age of this resin to the Turonian– Cenomanian, based on arthropod inclusions. Shi et al. [24], based on U-Pb dating of zircons from the volcaniclastic matrix of the amber, estimated the age of Burmese amber at 98.79 ± 0.62 Ma (earliest Cenomanian).

This published work and the nomenclatural acts it contains have been registered in ZooBank, the online registration system for the ICZN. The LSID for this publication is: urn:lsid:zoobank.org:pub:90A627F2-9ECB-4ACE-81FC-9AC006D90656. 

## 3. Results

### 3.1. Systematics Palaeontology

Order Diptera Linnaeus, 1758 [25].

Infraorder Bibionomorpha Hennig, 1948 [26].

Superfamily Sciaroidea Billberg, 1820 [27].

Family Keroplatidae Rondani, 1856 [4].

Genus *Paleoplatyura* Meunier, 1899 [9].

Type species: *Mycetobia macrocera* Loew, 1850 = *Paleoplatyura macrocera* (Loew, 1850) [9].

The genus includes one extant Holarctic species, *P. johnsoni*, and four fossil species, i.e., *P. macrocera*; *P. miae* sp. nov., *P. magnifica* sp. nov., *P. agnieszkae* sp. nov.

Diagnosis: Cross-vein r-m present; basal part of Mb clearly visible; cross-vein m-cu situated in between M_1+2_ and Cu; R_2+3_ oblique and ending in C, anal vein strong and reaching wing margin; gonostylus narrow and apically bifurcated.

### 3.2. Description of Amber Materials

***Paleoplatyura macrocera*** **(Loew, 1850).**

*Mycetobia macrocera* Loew, 1850—Baltic amber (about 42 MA).

Amended diagnosis: Antennae reach almost 2/3 of the wing length, probably 16 segmented; wing 2.5× longer than wide; Sc vein ends distinctly before Rb forks into Rs and R_1_; R_4+5_ almost 3× longer than Rs and almost equal in length to R_2+3+4+5_; cross-vein r-m short; M_1_ almost 4× longer than M_1+2_; m-cu distinctly beyond fork of Mb.

Remarks: *P. macrocera* is the type species of the genus *Paleoplatyura*. It was described on the basis of a single female inclusion in the Baltic amber. Unfortunately, the study is made more difficult by the missing holotype and the fact that despite the analysis of numerous specimens classified to Sciaroidea in various Baltic amber collections, no specimen belonging to this species was found. Therefore, the concept of the genus is based on the figure provided in the work of Meunier (1899, Figure 9 [9]). 

**Figure 1:**
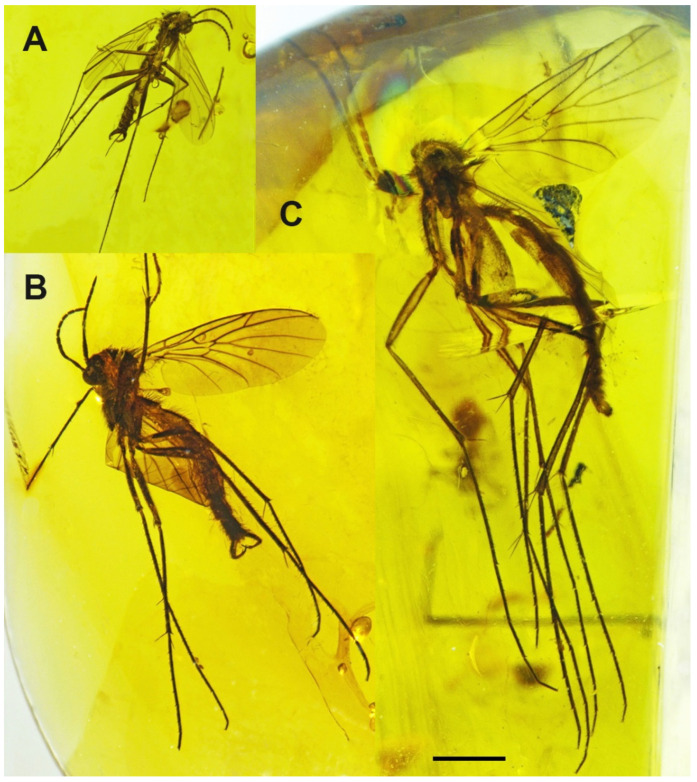
Habitus photographs of *Paleoplatyura agnieszkae* sp. nov. ((**A**) holotype), *P. miae* sp. nov. ((**B**) holotype), and *P. magnifica* sp. nov. ((**C**) holotype). Scale bar = 1 mm.

**Figure 2:**
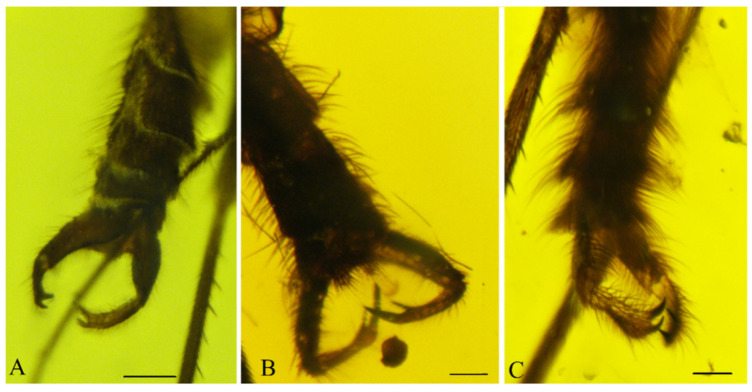
Male genitalia of *Paleoplatyura agnieszkae* sp. nov. (**A**), *P. miae* sp. nov. (**B**), and *P. magnifica* sp. nov. (**C**). Scale bar = 0.1 mm.

**Figure 3:**
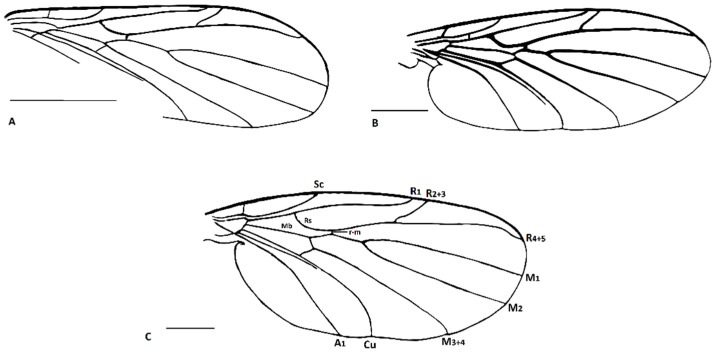
Wing of *Paleoplatyura agnieszkae* sp. nov. (**A**), *P. miae* sp. nov. (**B**), and *P. magnifica* sp. nov. (**C**). Scale bar = 0.5 mm.

**Figure 4:**
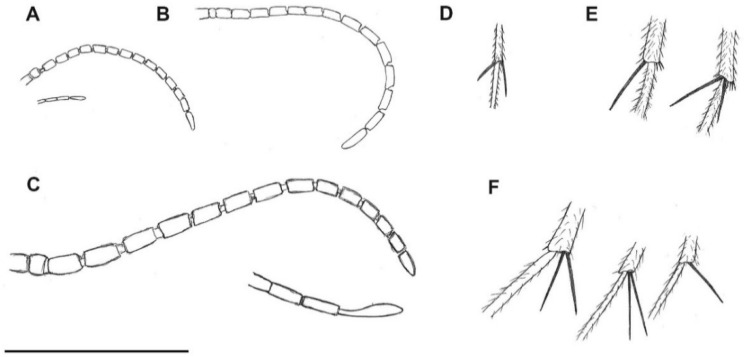
*Paleoplatyura agnieszkae* sp. nov., antenna (**A**) and middle leg (**D**); *P. miae* sp. nov., antenna (**B**); hind and middle leg (**E**); *P. magnifica* sp. nov., antenna (**C**), hind, middle, and foreleg (**F**). Scale bar = 1 mm.

**Figure 5:**
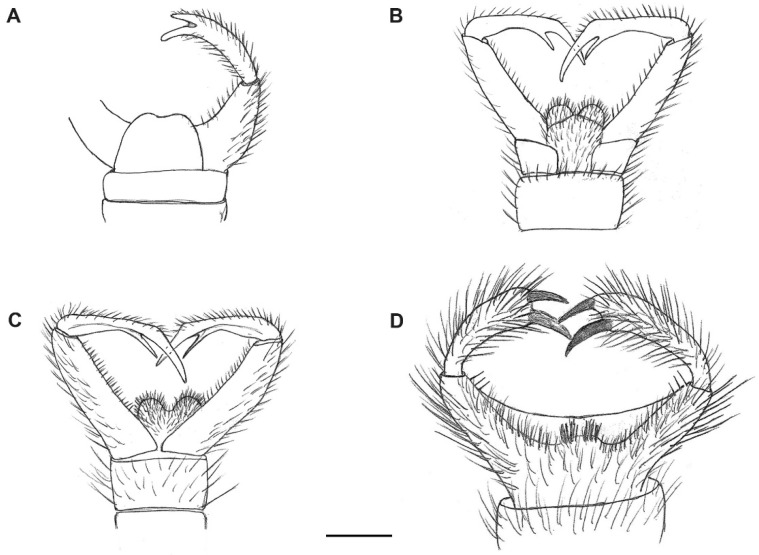
Male terminalia of *Paleoplatyura agnieszkae* sp. nov. (**A**), *P. miae* sp. nov. ((**B**) dorsal, (**C**) ventral), and *P. magnifica* sp. nov. (**D**). Scale bar = 0.1 mm.

***Paleoplatyura agnieszkae* sp. nov.**

(Figure 1A, Figure 2A, Figure 3A, Figure 4A,D and Figure 5A).

urn:lsid:zoobank.org:act:6BAC66C5-2B32-4C4D-8AD9-FA6729F8E6EC

Diagnosis: Sc ends just beyond Rb bifurcation into R_1_ and Rs; R_4+5_ more than 3× longer than Rs and about 2× longer than R_2+3+4_; M_1_ 2.5× longer than M_1+2_; gonostylus slightly longer than gonocoxites, strongly forked at end, the upper arm of bifurcation much shorter than the lower one; gonocoxite much expanded in basal part.

Etymology: The specific name, honours dr hab. Agnieszka Soszyńska-Maj, a well-known paleoentomologist, specializing in fossil Diptera and Mecoptera.

Material examined: Holotype (male), No. MP/4288, Burmese amber; deposited in the collection of ISEA PAS.

Description: Wing length 1.6 mm, width 0.6 mm (Figure 1A). Head: Antennae with 16 segments; scapus wide and barrel-shaped; pedicel oval; flagellomeres almost 1.5× longer than its width, and the last segment almost 3× longer than its width; palpi short (Figure 4A). Wing almost 3× longer than its width; Sc ends just beyond fork of Rb into R_1_ and Rs; R_1_ ends opposite half the length of R_2+3_, near tip of R_2+3_; R_2+3_ distinctly waved at mid-length; R_4+5_ more than 3× longer than Rs and 2× longer than R_2+3+4_; r-m short, equals ⅕ of length of Rs, located in ⅓ length of M_1+2_; Mb present, distinctly visible; M_1_ about 2.5× longer than M_1+2_; m-cu just beyond fork of Mb, located between M_3+4_ and Cu; Cu slightly arched; pseudovein (ps) clearly visible; A_1_ visible only in basal part (Figure 3A). Legs: foreleg with a single spur almost 3.5 times the width of tibia; hind and middle leg with two spurs of unequal length (Figure 4D). Hypopygium; Gonocoxite broad, greatly expanded in basal part (length about: 0.12 mm); gonostylus slightly longer than gonocoxites (length about: 0.14), strongly forked at end, the upper arm of bifurcation much shorter than the lower one (Figure 2A and Figure 5A). 

***Paleoplatyura miae* sp. nov.**

(Figure 1B, Figure 2B, Figure 3B, Figure 4B,E and Figure 5B,C).

urn:lsid:zoobank.org:pub:90A627F2-9ECB-4ACE-81FC-9AC006D90656

Diagnosis: Sc ends distinctly beyond Rb bifurcation at R_1_ and Rs; R_4+5_ 3× longer than Rs and almost 2× longer than R_2+3+4_; M_1_ about 4.5× longer than M_1+2_; gonostylus almost equal in length to gonocoxites, strongly bifurcated at end, the upper arm of bifurcation almost 2× longer than lower one.

Etymology: The specific epithet is given after name of the granddaughter, Mia, of one of the authors (WK).

Material examined: Holotype (male), No. MP/4075, Burmese amber; deposited in the collection of the ISEA PAS.

Description: Wing length 2.9, width 1.2 (Figure 1B). Head. Antennae with 16 segments; scapus tubular; pedicel short and oval; flagellomeres increasing in length and the last segment nearly 5× longer than its width; palpi short (Figure 4B). Thorax: Wing about 2.7 times longer than its width; Sc ends beyond fork of Rb into R_1_ and Rs; approximately opposite ⅓ of length of Rs; R_1_ ends opposite half the length of R_2+3_, near tip of R_2+3_; R_2+3_ nearly straight; R_4+5_ more than 3× longer than Rs and almost 2× longer than R_2+3+4_; r-m short, equals ⅙ of length of Rs, located distinctively before middle of length of M_1+2_; Mb present, clearly visible; M_1_ more than 4× longer than M_1+2_; m-cu just beyond fork of Mb, located between M_3+4_ and Cu; Cu at end strongly waved; pseudovein (ps) clearly visible; A_1_ with apical half strongly bent to wing margin (Figure 3B). Legs: foreleg and midleg with single spur that are nearly 3.5 times the width of tibia; hind leg with two spurs of unequal length (Figure 4E). Hypopygium: Gonocoxites long and narrow (length about: 0.28 mm); gonostylus almost equal in length to gonocoxites (length about: 0.27 mm), forked at end, the upper arm of bifurcation almost 2× longer than the lower (Figure 2B and Figure 5B,C).

***Paleoplatyura magnifica* sp. nov.**

(Figure 1C, Figure 2C, Figure 3C, Figure 4C,F and Figure 5D).

LSID urn:lsid:zoobank.org:act: E0E4BF32-60A9-4C69-86C4-390591FB6B30

Diagnosis: Wing length: 3.8 mm. Sc ends far beyond Rb bifurcation to R_1_ and Rs; R_4+5_ more than 3× longer than Rs and about 2× longer than R_2+3+4_; M_1_ 3× longer than M_1+2_; gonostylus almost equal in length to gonocoxite, at end bifurcated, processes of equal length, strongly sclerotized; gonocoxite broad and much expanded in basal part.

Etymology: The specific epithet is given to emphasize the large size of the specimen (from the Latin *magnifico*/feminine *magnifica*/, meaning magnificent or gorgeous).

Material examined: Holotype (male), No. MP/4076—Burmese amber; deposited in the collection of the ISEA PAS.

Description: Wing length 3.8 mm, width 1.8 mm (Figure 1C). Head: antennae with 16 segments; scapus tubular; pedicel short and oval; flagellomeres nearly 2× longer than their width; palpi relatively long, segments of nearly the same length (Figure 4C). Wing about 2⅓× longer than its width; Sc ends far beyond fork of Rb into R_1_ and Rs, opposite ⅔ of length of Rs; R_1_ ends opposite half the length of R_2+3_, near the tip of R_2+3_; R_2+3_ distinctly waved at basal part; R_4+5_ more than 3× longer than Rs and about 2× longer than R_2+3+4_; r-m short, equals ⅛ of length of Rs, located close to middle of length of M_1+2_; Mb present, clearly visible; M_1_ 3× longer than M_1+2_; m-cu more than its own length beyond fork of Mb, situated between M_3+4_ and Cu; Cu at end strongly bent; pseudovein (ps) clearly visible; A_1_ slightly wavy at the middle of its length (Figure 3C). Legs: front leg with a single spur, middle and hind leg with two spurs of nearly the same length and nearly 3× longer than the width of tibia (Figure 4F). Hypopygium: Gonocoxite broad and greatly expanded in the basal part; gonostylus almost equal in length to gonocoxite, forked at the end, processes of equal length, strongly sclerotized (Figure 2C and Figure 5D). 

### 3.3. Key to Fossil Species of Paleoplatyura 

1. Sc ends before Rb forks into R_1_ and and Rs …………… *P. macrocera* (Loew, 1850)  - Sc ends after Rb forks into R_1_ and and Rs ………………………………… 22. Pattern of tibial spurs 1:1:2 ………………………………………… *P. miae* sp. nov.  - Pattern of tibial spurs 1:2:2 ……………………………………… ………… 33. Costa produced to less than ¼ of the distance between tips of R_4+5_ and M_1_; tibial spurs of same length; apical processes strongly sclerotized ………… *P. magnifica* sp. nov.  - Costa produced to about ¼ of the distance between tips of R_4+5_ and M_1_; tibial spurs of different length; gonostylus narrow throughout its length; apical processes not sclerotized ……………………………………………………… *P. agnieszkae* sp. nov.

## 4. Discussion

The type species of the genus *Paleoplatyura*, *P. macrocera*, from Baltic amber, differs from all congeners in Burmese amber by the short vein Sc, which ends clearly before the bifurcation of Rb into R_1_ and Rs. Surprisingly, the Burmese amber species of *Paleoplatyura* are thus more similar in this respect to the extant species *P. johnsoni* than to the Baltic amber type species. However, the relative length of Sc also differs among various species of *Macrocera* Meigen, 1803 [13], and similar variation is known in some genera of Mycetophilidae, so that this character appears as species-specific rather than of fundamental phylogenetic importance. We thus prefer to maintain the concept of *Paleoplatyura* as defined in this paper; which means including intrageneric variation of the length of Sc, considering the well-known fact that higher taxonomic categories, like genus or subfamily, are usually more or less subjective, and their concept (breadth) may differ, even within one family. The subjective concept of genera, with different breadth defined by various authors, may also be a key to the understanding why Mesozoic genera, such as *Paleoplatyura,* are (seemingly) present in both the Tertiary and extant fauna.

The presence of extant genera in the Mesozoic fauna is a remarkable phenomenon itself. Recent studies of Burmese amber inclusions increasingly indicate the origin of modern genera as early as in the Cretaceous. Examples of extant insect genera found in the Mesozoic are well documented, though not common. Grimaldi and Cumming [28] stated that *Apalocnemis canadambris* Grimaldi and Cumming [28] (family Empididae), was the only species out of 49 species studied within their paper on Cretaceous ambers Brachycera belonging to an extant genus. In Diptera, several other similar cases are known from Burmese amber, e.g., *Antocha lapra* Podenas and Poinar [29], and *Helius lebanensis* Kania, Krzemiński and Azar [30], from the family Limoniidae, or *Nemopalpus quadrispiculatus* Stebner et al. [31], and *Phlebotomus vetus* Stebner et al. [31], from the family Psychodidae. In some cases, a recent genus serves only as a “wastebasket taxon”, to tentatively place a problematic species in a described genus, as is the case of some genera of Mycetophilidae in Cretaceous ambers [32]. A similar situation is reported in [33], e.g., for the click-beetle genus *Elater* Linnaeus [25]. Some taxa of Diptera appeared even earlier, e.g., the genus *Protanyderus* Handlirsch, 1909 (family Tanyderidae), in the Upper Jurassic of Mongolia [34].

The extant species *Paleoplatyura johnsoni*, which unambiguously belongs to *Paleoplatyura*, was described from North America, and recently found also in Europe (southern Italy, see [12]). It is a large species (wing length is 8 mm), significantly larger than the fossil congeners, with the wings strongly marked, and relatively short antennae, only slightly longer than the head and thorax together. Surprisingly, there are no new specimens of *Paleoplatyura* available from Baltic amber. In contrast, several specimens from this genus, belonging to the three species described in this paper, have been found in Burmese amber. A major problem in the study of fossil Keroplatidae, is the loss of most of the holotypes of previously described species, especially from Baltic amber, and the lack of a recent, comprehensive morphological study of Keroplatidae belonging to the tribe Orfeliini. Additionally, the concept of the tribes Keroplatini and Orfeliini appears as outdated in the light of modern molecular studies [1].

A similar wing venation and structure of the male terminalia as in *Paleoplatyura* are found in the genus *Asynaphleba* Matile, 1974 [35], containing a single South African extant species, which differs from *Paleoplatyura* only by the absence of the vein Mb and by shorter anal vein, not reaching the wing margin. Thus, it seems that the presence of both Mb and the cross-vein r-m are good diagnostic characters for the genus *Paleoplatyura*. Taking into consideration only a distinct vein Mb, as a clear plesiomorphic character state, it is not unique to *Paleoplatyura* within keroplatids, but it is present also in several genera of Macrocerinae, including the species-rich genus *Macrocera*, and also in the South African genus *Schizocyttara* Matile, 1974 [35], which was shown to be closely related to *Paleoplatyura johnsoni* by Mantič et al. [1], although it lacks cross-vein r-m. Both Mb and cross-vein r-m are well developed also in the genus *Arachnocampa* Edwards, 1924 [36], from the most plesiomorphic keroplatid subfamily Arachnocampinae, which, however, differs from *Paleoplatyura* in some other characters, such as the absence of R_2+3_ and different proportion of wing veins.

## 5. Conclusions

Genus *Paleoplatyura* represents one of the most ancient and plesiomorphic genera of Keroplatidae, with the most complete wing venation representing a ground plan within the family (together with genus *Arachnocampa*). This group possesses also the most plesiomorphic structure of male terminalia, which appears to be simple with long and apically forked gonostyli. Molecular data indicate a close relationship of *Paleoplatyura* with Macrocerinae [1]; additionally, if we compare the wing of *Paleoplatyura* with that of *Macrocera*, we can see many similarities, confirming the evolutionary trend to the reduction of radio-medial cross-vein to the so-called radio-medial fusion, typical of most keroplatids, together with prolongation of the antennae in *Macrocera* and elimination of some wing veins in Lygistorrhininae. The basal part of medial vein is still retained in most Macrocerinae, while it is absent in almost all species of the more apomorphic subfamilies of Keroplatinae and Lygistorrhininae. The overall structure of male terminalia is also very similar in *Paleoplatyura* and most Macrocerinae.

## Data Availability

Not applicable.
